# Antioxidant, Mineralogenic and Osteogenic Activities of *Spartina alterniflora* and *Salicornia fragilis* Extracts Rich in Polyphenols

**DOI:** 10.3389/fnut.2021.719438

**Published:** 2021-08-18

**Authors:** Vânia P. Roberto, Gwladys Surget, Klervi Le Lann, Sara Mira, Marco Tarasco, Fabienne Guérard, Nathalie Poupart, Vincent Laizé, Valérie Stiger-Pouvreau, M. Leonor Cancela

**Affiliations:** ^1^Centre of Marine Sciences (CCMAR), University of Algarve, Faro, Portugal; ^2^University of Brest, CNRS, IRD, Ifremer, LEMAR, IUEM, Plouzané, France; ^3^S^2^-AQUA - Sustainable and Smart Aquaculture Collaborative Laboratory, Olhão, Portugal; ^4^Faculty of Medicine and Biomedical Sciences, University of Algarve, Faro, Portugal; ^5^Algarve Biomedical Center, Faro, Portugal

**Keywords:** antioxidant, phenolic compounds, mineralization, osteogenesis, osteoporosis, marine plants

## Abstract

Osteoporosis is an aging-related disease and a worldwide health issue. Current therapeutics have failed to reduce the prevalence of osteoporosis in the human population, thus the discovery of compounds with bone anabolic properties that could be the basis of next generation drugs is a priority. Marine plants contain a wide range of bioactive compounds and the presence of osteoactive phytochemicals was investigated in two halophytes collected in Brittany (France): the invasive *Spartina alterniflora* and the native *Salicornia fragilis*. Two semi-purified fractions, prepared through liquid-liquid extraction, were assessed for phenolic and flavonoid contents, and for the presence of antioxidant, mineralogenic and osteogenic bioactivities. Ethyl acetate fraction (EAF) was rich in phenolic compounds and exhibited the highest antioxidant activity. While *S. fragilis* EAF only triggered a weak proliferative effect *in vitro, S. alterniflora* EAF potently induced extracellular matrix mineralization (7-fold at 250 μg/mL). A strong osteogenic effect was also observed *in vivo* using zebrafish operculum assay (2.5-fold at 10 μg/mL in 9-dpf larvae). Results indicate that polyphenol rich EAF of *S. alterniflora* has both antioxidant and bone anabolic activities. As an invasive species, this marine plant may represent a sustainable source of molecules for therapeutic applications in bone disorders.

## Introduction

Osteoporosis is a metabolic disease affecting 200 million people worldwide (1). It is characterized by a gradual loss of bone that results from an unbalanced remodeling process, where bone formation by osteoblasts does not compensate bone resorption by osteoclasts (2). Disease progression is characterized by an altered bone microarchitecture with an increased risk of fracture, often resulting in a reduced well-being and increased mortality (3). Therapies currently available mainly relies on antiresorptive drugs, e.g., bisphosphonates and anti-RANKL antibody (Denosumab), which have only reasonable efficiency and limited long term usage due to their side effects (2, 4). Therapeutic or preventive strategies capable of promoting bone formation are limited and Teriparatide is the only bone anabolic treatment currently licensed for osteoporosis (5). Current scenario highlights the urging need for the discovery of novel anabolic compounds with pharmaceutical or nutraceutical value.

As a multifactorial and age-related disease, osteoporosis arises from the interplay of several factors that contribute for increasing the risk of disease, including genetic, epigenetic and environmental factors, diet and lifestyle (4, 6, 7). In the last years, oxidative stress has emerged as a critical lifestyle risk factor associated with the loss of bone mineral density (8, 9). In this regard, antioxidants have been reported to positively impact on bone formation and osteoblast differentiation, while limiting bone resorption and osteoclastogenesis (9–14). All these studies indicate that targeting the production of reactive oxygen species (ROS) may be a relevant approach for future therapies related to bone diseases.

Antioxidants are known to prevent cellular damages that could result from chemical reactions initiated to counteract the excessive production of ROS (15, 16). They are central to several physiological processes, from DNA damage, cell apoptosis, inflammation and immunity regulation to aging. In this aspect, unbalanced levels of antioxidants and ROS have been associated with several human pathologies, including bone diseases (9, 17). Thus, it has been proposed that a diet rich in natural antioxidants, e.g., from vegetables and fruits, or complemented with food supplements containing antioxidants, can be used as a preventive approach for bone diseases such as osteoporosis.

In the past three decades, several studies have shown that marine plants are one of the richest and most promising sources of bioactive primary and secondary metabolites (18). As they need to protect themselves against abiotic and biotic factors (generators of intracellular ROS), marine plants produce a high diversity of antioxidant metabolites to counterbalance the production of intracellular ROS (18, 19). This is particularly true for invasive species that need to develop a strong phenotypic acclimatation in order to colonize vast habitats with variable environmental characteristics (20). Among these metabolites, phenolic compounds are of particular interest as they exhibit multiple biological activities (21, 22). They can be simple molecules, such as phenolic acids, or polymerized compounds such as polyphenols. Flavonoids account for most of the phenolic compounds (23) and are well-known for their antioxidant activities (24–26). Because the synthetic compounds used as antioxidants in the industry may be toxic and carcinogenic (27, 28), there is a growing interest in natural antioxidants, as those from macrophyte species, for applications as active ingredient in the food, cosmetic and pharmaceutical industries (21, 25). Phenolic compounds also have properties that are not directly related to their biological role in the organism from which they are extracted. In this regard, a bone anabolic effect of phenolic compounds has been reported (22, 29–32), suggesting that these compounds could be used in the prevention of low bone mineral density disorders such as osteoporosis (33). However, the role of phenolic compounds and antioxidant molecules in bone metabolism and bone diseases remains to be fully understood.

In the present study, we assessed the presence of phenolic compounds in extracts from the Poaceae *Spartina alterniflora* Loisel and the Amaranthaceae *Salicornia fragilis* Ball & Tutin, synonym for *Salicornia procumbens* Smith (34). The first species is invasive in Brittany and available in large quantity at many sites, thus could be useful for valorization. The second species is endogenous to Brittany and belongs to the same genus of *Salicornia ramossissima*, which has been shown previously to exhibit high antioxidant activity (22). We then compared both species and evaluated their potential use as antioxidants for applications in bone-related diseases.

## Materials and Methods

### Biological Samples

Both the invasive plant *Spartina alterniflora* (Cyperales, Poaceae) and the native plant *Salicornia fragilis* (Caryophyllales, Amaranthaceae) were sampled from the marshes of the Elorn River (48°24'26.10” N, 4°20'47.82” W; Le Relecq Kerhuon, 29) and the Ria du Conquet (48°21'47.71” N, 4°45'21.23” W; Le Conquet, 29), respectively. Plant material was carefully sorted and identified according to Murakeözy et al. (35). It was rinsed briefly under running tap water before being frozen (−24°C) and freeze-dried (CHRIST Beta 1-8 LD). The samples were then ground to powder using a household grinder and stored in the dark at room temperature.

### Extraction and Liquid-Liquid Semi-purification Process

Crude extract (CE) was prepared by mixing 30 g of plant powder with 300 mL of an ethanol-water mixture (1:1, v:v, EtOH 50) in the dark at 40°C for 2 h under rotary agitation (200 rpm). Macerate was centrifuged at 3,000 × g at 10°C for 10 min, and supernatant was collected. Pellet was re-extracted twice with 300 mL of EtOH 50 in the dark at 40°C for 1 h under rotary agitation (200 rpm), and centrifuged. Pooled supernatants (900 mL in total) were evaporated at 40°C under vacuum to reach 100 mL of CE which was frozen and freeze-dried. Then CE, dissolved in distilled water, was subjected to a liquid–liquid purification process to concentrate the phenolic compounds using a methodology already described (36, 37). Briefly, the first step involved three dichloromethane washes; the second step consisted in rinsing the aqueous phase (containing polyphenols) with acetone and then with ethanol; the third step comprised three ethyl acetate rinses that gave the aqueous (AF) and the ethyl acetate (EAF) fractions. At the end of the purification procedure, each fraction was evaporated using a rotary evaporator, dissolved in water, frozen, freeze-dried, and stored until further used.

### Total Phenolic Content and Flavonoid Content

An aliquot of each dry extract (CE, EAF and AF) was solubilized in demineralized water at a concentration of 10 g/L. Dilutions from 0.005 to 1 g/L were tested in triplicate. All colorimetric tests were carried out in 96-well microplates (Greiner Bio-One) and absorbance was determined in a microplate spectrophotometer (Multiscan MS, Labsystems). All chemicals were from Sigma-Aldrich unless otherwise indicated. The total phenolic content was quantified using the Folin–Ciocalteu procedure adapted to microplates (38). Each well was filled with 20 μL of extract, 130 μL of distilled water, 10 μL of Folin–Ciocalteu reagent and 40 μL of sodium carbonate (200 g/L). Plates were incubated at 70°C for 10 min and then placed on ice to stop the chemical reaction. Absorbance was measured at 620 nm. Phenolic content was quantified based on gallic acid standard curve and results were expressed in mg gallic acid g^−1^ of dried extract (DW). Flavonoid content was determined using a method adapted from Dewanto et al. (39). Each well was filled with 25 μL of extract, 100 μL of distilled water and 10 μL of sodium nitrite (5%; w/v). Plates were incubated for 5 min at room temperature. Then, 20 μL of aluminum chloride (5%; w/v) were added and plates incubated for another 5 min. Reaction was stopped with 50 μL of 1 M sodium hydroxide. Absorbance was read at 492 nm. Flavonoid content was quantified based on a catechin (flavanol) standard curve and results were expressed as mg catechin g^−1^ DW.

### Biological Activities

#### Antioxidant Activity

Activities covering different antioxidant mechanisms (40) were evaluated in this study. Radical scavenging and antioxidant activities were measured with four colorimetric analyzes: 2,2-diphenyl-1-picrylhydrasyl (DPPH) assay, ferric reducing antioxidant power (FRAP) assay, β-carotene bleaching test (BCBT) and oxygen radical absorbance capacity (ORAC) test.

##### DPPH Assay

Radical scavenging activity was evaluated by the DPPH assay, which is based on the reduction of the free radical DPPH by antioxidant molecules such as phenolic compounds. Each well was filled with 22 μL of extract and 200 μL of DPPH solution at 25 mg/mL, freshly prepared within 48 h. Plates were incubated for 60 min in the dark and at room temperature. Absorbance was read at 540 nm. Negative controls are distilled water and ethanol, while positive controls are 2,3-t-butyl-4-hydroxyanisole (butylated hydroxylanisole, BHA) and ascorbic acid (vitamin C). The radical scavenging activity was determined from the IC_50_ value (mg/mL). A low IC_50_ value indicates a high radical scavenging activity. This analysis was carried out in triplicate for each fraction.

##### FRAP Assay

FRAP assay is based on a redox reaction between phenolic compounds and transition metal ions like ferric ions. Method was adapted from (38). Each well was filled with 25 μL of extract, 25 μL of 0.2 M sodium phosphate buffer (pH 6.6), and 25 μL of 1% ferricyanide potassium. After homogenization, plates were incubated at 50°C for 20 min. Reaction was stopped on ice by adding 25 μL of 10% trichloroacetic acid and 100 μL of distilled water. Absorbance was measured at 620 nm, then 20 μL of 0.1% iron chloride was added. After 10 min, absorbance was measured again. Vitamin C and BHA were used as positive controls. FRAP activity was determined from the EC_50_ value (mg/mL). A low EC_50_ value indicates a high reducing power. This analysis was carried out in triplicate for each sample.

##### β-Carotene Bleaching Test

Antioxidant activity measured using the β-carotene bleaching method (41, 42) was determined following the protocols published by (38, 43). Briefly, 2 mL of a solution of β-carotene in chloroform (0.1 mg/mL) was added to round-bottom flasks containing 20 mg of linoleic acid and 200 mg of Tween 40. After evaporation with a rotavapor, oxygenated distilled water (50 mL) was added and the mixture was shaken to form a liposome solution. This mixture was added to 12 μL of extracts, positive controls (α-tocopherol, Trolox, and BHA) or negative controls (distilled water and ethanol). Absorbance was measured at 450 nm immediately (*t* = 0 min) and after 2 h at 50°C (*t* = 120 min). All samples were assayed in triplicate. Antioxidant activity coefficient (AAC700 in mg per mL) was calculated as described Le Lann et al. (43). A low AAC700 indicates a strong antioxidant activity.

##### Oxygen Radical Absorbance Capacity (ORAC) Test

ORAC value (44) was determined by InVivo Labs (Vannes, France) for the most active fraction using a protocol adapted from Cao et al. (45). Results were expressed as μmol Trolox equivalent (Te) mg^−1^ DW and were compared to ascorbic acid.

#### Cytotoxic Activity

Gilthead seabream (*Sparus aurata*) bone-derived cell line VSa13 were maintained in Dulbecco's modified Eagle medium (DMEM) with 10% FBS (fetal bovine serum, Sigma-Aldrich), 1% penicillin–streptomycin, 1% L-glutamine and 0.2% fungizone (all from GIBCO, Thermo Fisher Scientific) at 33 °C in a 10% CO_2_-humidified atmosphere (46, 47). Cells were sub-cultured 1:4 twice a week using trypsin-EDTA solution (0.2% trypsin, 1.1 mM EDTA, pH 7.4, GIBCO, Thermo Fisher Scientific). For the cytotoxicity assay, 10^4^ cells/well were seeded in 96-well plates and further cultured until confluence. Culture medium was replaced with fresh medium containing either the vehicle (control) or plant extracts and was renewed every 3–4 days. Dry extract/fractions were dissolved in distilled water (AF), 50% ethanol (CE) or 100% ethanol (EAF) according to their polarity to prepare 1,000 × stock solutions. These were then added to the culture medium to achieve the working concentrations ranging from 0.05 to 250 μg/mL. Supplemented culture medium was 0.2-μm filtered before applied to the cell culture. Cell viability was assessed after 9 and 18 days of exposure using the Cell Proliferation Kit XTT (AppliChem).

#### Proliferative Activity

VSa13 cells were cultured as described above and seeded in 96-well plates at a density of 1.5 × 10^3^ cells per well. After 24 h, culture medium was replaced with fresh medium containing either the vehicle (control) or plant extracts and renewed every 3–4 days. Extracts and fractions were dissolved in distilled water (AF), 50% ethanol (CE) or 100% ethanol (EAF) to achieve final concentrations ranging from 0.05 to 100 μg/mL, as described above. Cell proliferation was determined after 9 days of exposure using the Cell Proliferation Kit XTT.

#### Mineralogenic Activity

VSa13 cells were cultured as described above and seeded in 24-well plates at a density of 5 × 10^4^ cells per well. Extracellular matrix (ECM) mineralization was induced in confluent cultures by supplementing culture medium with L-ascorbic acid (50 μg/mL), β-glycerophosphate (10 mM) and calcium chloride (4 mM). Extract and fractions dissolved in distilled water (AF), 50% ethanol (CE) or 100% ethanol (EAF) were added to the culture medium with final concentrations ranging from 0.05 to 250 μg/mL, as described above. After 17 days of culture, mineral deposition was revealed through alizarin red S (AR-S; Sigma-Aldrich) staining and quantified by spectrophotometry (48).

#### Osteogenic Activity

Broodstock of adult zebrafish (AB strain) were maintained in a water recirculating system (ZebTEC, Tecniplast) under the following conditions: temperature 28 ± 0.1°C, pH 7.5 ± 0.1, conductivity 700 ± 50 μS, ammonia and nitrites lower than 0.1 mg/L, nitrates at 5 mg/L, and a 10:14 h dark-light photoperiod. Conductivity and pH were stabilized in fish water by adding Instant Ocean salt mixture and sodium bicarbonate to reverse osmosis treated water. Sexually mature zebrafish were crossed following an in-house breeding program using a 5:3 female to male ratio (different groups of breeders were used). Fertilized eggs were transferred into a 1-L container with static water conditions. Methylene blue (0.0002% w/v) was added to prevent fungal growth. Non-fertilized, asymmetrical, vesicle-containing, or damaged eggs were discarded.

Zebrafish larvae were exposed to increasing concentrations of *S. alterniflora* EAF (1, 5 and 10 μg/mL), control (0.1% ethanol) or calcitriol (10 pg/mL, Sigma-Aldrich) from 9 to 11 days post-fertilization (dpf) as previously described (32). From 5 dpf, larvae were fed twice a day with freshly hatched *Artemia* nauplii (AF480 strain, INVE Aquaculture). The most effective concentration of EAF was tested in the optimized zebrafish operculum system (49). Briefly, larvae were exposed to EAF (10 μg/mL), control (0.1% ethanol) or calcitriol (10 g/mL) from 3 to 6 dpf, in a 6-well plate (15 larvae in 10 mL of water) placed in the dark, to avoid photodegradation of the compounds, at 28.5°C. Treatment was renewed (70% of the total volume) once a day. After 72 h of exposure, larvae were sacrificed with a lethal anesthesia of 168 μg/mL of tricaine (MS-222; Sigma-Aldrich), and then stained with 0.01% of alizarin red S at room temperature for 15 min, washed twice with Milli-Q water for 5 min and imaged (50). The area of the head was used to normalize the area of the operculum of each larvae [adapted from (49, 51)].

### One-Dimensional Proton Nuclear Magnetic-Resonance

1D ^1^H NMR spectra were acquired at 25°C on a Bruker Avance 400 MHz spectrometer equipped with an inverse triple resonance broadband (TBI) 5-mm probe ^1^H/(BB)/^31^P. NMR analyses were recorded on samples dissolved in 700 μL of 100% D_2_O (deuterium oxide) for the crude extract and the aqueous fraction and of 100% MeOD (deuterated methanol) for the ethyl acetate fraction, according to standard Bruker program acquisitions. Spectra were obtained using a 2 s delay and scan 30-degree angle pulse. Chemical shifts are expressed in ppm relative to trimethylsilyl propionate (TSP) as an external reference.

### Statistical Analysis

All tests were carried out in triplicate and results were expressed as means ± standard deviation (SD). Phenolic content and antioxidant data were analyzed with RStudio (v.0.95.263) integrated R (v.2.12.0). Data not homogenous for variances were analyzed using the non-parametric Kruskal–Wallis test at a significance level of 95%, followed by the Behrens–Fisher non-parametric multiple comparisons test (npmc package). Correlations between measured biological variables were tested to illustrate correlations between the extract composition and biological activities. This analysis was based on the Spearman method (Hmisc package). Cytotoxicity, proliferation, mineralization and osteogenic data were analyzed through one-way ANOVA followed by Dunnett's multiple comparison test (*p* < 0.05) using Prism version 8.2.1 (GraphPad Software, Inc. La Jolla, CA).

## Results

### Content of Phenolic Compounds and Flavonoids

The biological material – powdered thalli from *Spartina alterniflora* and *Salicornia fragilis* – was submitted to an extraction and liquid-liquid semi-purification process to prepare an hydroalcoholic crude extract (CE) and two semi-purified fractions: an aqueous fraction (AF) and an ethyl acetate fraction (EAF). EAF of both species were rich in phenolic compounds, although Total Phenolic Content (TPC) was more than 2 times higher in *S. fragilis* EAF (Table 1). TPC levels were much lower in CE and AF but similar in both species. Phenolic compounds were 13 times higher in *S. fragilis* EAF compared to CE (262.02 ± 1.49 vs. 20.18 ± 0.36 mg GAE g^−1^ DW, respectively) but only 5 times in *S. alterniflora* EAF (117.74 ± 3.42 vs. 21.96 ± 0.62 mg GAE g^−1^ DW, respectively; Table 1). Flavonoids were only detected in *S. fragilis* (Table 1), where they represent the totality of the phenolic compounds in the three extracts/fractions in study. Highest flavonoid content was detected in *S. fragilis* EAF (285.09 ± 2.81 mg C g^−1^ DW). In contrast, flavonoids were not detected in *S. alterniflora* EAF but represented 50–60% of the phenolic compounds in CE and AF (Table 1). The results obtained for the ethyl acetate fraction of *S. alterniflora* could not be explored due to interference from the strong coloration of this fraction.

**Table 1:** Phenolic and flavonoid contents and antioxidant activities in extracts of *Spartina alterniflora* and *Salicornia fragilis*.

		**Phenolic content**	**Flavonoid content**	**β-carotene**	**DPPH scavenging activity**	**FRAP assay**
		**(mg GAE/g DW)**	**(mg C/g DW)**	**(IC_**50**_ g/L)**	**(IC_**50**_ g/L)**	**(EC_**50**_ g/L)**
*S. alterniflora*	CE	21.96 ± 0.62	13.76 ± 1.34	3.82 ± 0.48	0.365 ± 2.2E-03	4.160 ± 0.152
	EAF	**117.74** **±** **3.42**	*n.d*.	**0.39** **±** **0.06**	**0.077** **±** **2.1E-04**	**1.081** **±** **0.139**
	AF	19.05 ± 0.92	10.10 ± 1.34	7.19 ± 1.50	0.337 ± 3.6E-02	3.635 ± 0.059
*S. fragilis*	CE	20.18 ± 0.36	21.93 ± 1.12	2.24 ± 0.34	0.177 ± 5.8E-04	2.953 ± 0.039
	EAF	**262.02** **±** **1.49**	**285.09** **±** **2.81**	**0.32** **±** **0.02**	**0.013** **±** **1.2E-04**	**0.239** **±** **0.001**
	AF	15.65 ± 0.27	16.12 ± 1.12	3.37 ± 0.51	0.222 ± 3.5E-03	3.562 ± 0.148
Positive controls	BTH	–	–	0.0067 ± 4.0E-04	–	–
	Ascorbic acid	–	–	–	0.005 ± 8.9E-05	0.071 ± 0.001
	α-tocopherol	–	–	–	0.011 ± 7.9E-05	0.358 ± 0.020
	BHA	–	–	–	0.010 ± 2.2E-04	0.091 ± 0.001
	Trolox	–	–	–	0.007 ± 1.3E-04	0.171 ± 0.003

*Commercial standards (ascorbic acid, α-tocopherol, BHA and Trolox) were used as positive controls. Results are expressed as means ± standard deviation (n = 3). Values in bold indicate the most active fractions. GAE, gallic acid equivalent; C, catechin; DW, dry weight; DPPH, 2,2-diphenyl-1-picrylhydrazyl; IC_50_, half maximal inhibitory concentration; EC_50_, half maximal effective concentration; CE, crude extract; EAF, ethyl acetate fraction; AF, aqueous extract; BHA, butylated hydroxyanisole; BTH, butylated hydroxytoluene; n.d., not detected*.

### Antioxidant Activity

The antioxidant capacities of the six extracts under study as well as positive controls were assessed by BCBT, DPPH and FRAP assays (Table 1). Overall, AF presented the lowest antioxidant capacity while EAF of both species exhibited the highest antioxidant activities regardless of the assay. According to DPPH and FRAP assays, the antioxidant activities of *S. fragilis* EAF (0.013 ± 1.2E-04 and 0.239 ± 0.001, respectively) were higher than those of *S. alterniflora* EAF (0.077 ± 2.1E-04 and 1.081 ± 0.139, respectively), with values closer to those of the positive controls (Table 1). Concerning the BCBT assay, EAF is still the fraction presenting the best activity for both species, with, respectively, 0.39 ± 0.06 and 0.32 ± 0.02 mg/L in *S. alterniflora* and *S. fragilis*, (Table 1). Nevertheless, these fractions are less active than the standard BHT. Both species presented higher antioxidant activities with the DPPH assay. Our data suggest that the metabolites present in the EAF of both species have high antioxidant activities although the specific phenolic compounds should be different since *S. fragilis* EAF fraction is rich in flavonoids and two times richer in phenolic compounds (Folin-Ciocalteu procedure) than in *S. alterniflora* EAF (Table 1).

### Proliferative and Mineralogenic Activities

Since the aim of this study was to highlight compounds that would promote bone formation and mineralization, CE, AF and EAF of *S. fragilis* and *S. alterniflora* were first evaluated for their capacity to enhance bone cell proliferation and mineralization. Sub-toxic doses were determined by exposing VSa13 cells to a wide range of concentration for 9 days (endpoint for cell proliferation assay) and 18 days (endpoint for ECM mineralization assay). Cell survival rates in cultures exposed to the different extracts were not different from those in control cultures for most of the concentrations tested. However, exposure to the highest concentrations of AF and CE slightly reduced survival rates at 9 and 18 days (Supplementary Figures 1, 2). Interestingly, EAF of both species did not affect cell survival at neither 9 nor 18 days. Given the weak cytotoxic effect of the 6 extracts, CE and AF were used at 0.5, 5, 50, and 100 μg/mL and EAF at 0.05, 0.5, 5, and 50 μg/mL in the subsequent proliferation and mineralization assays.

**Figure 1:**
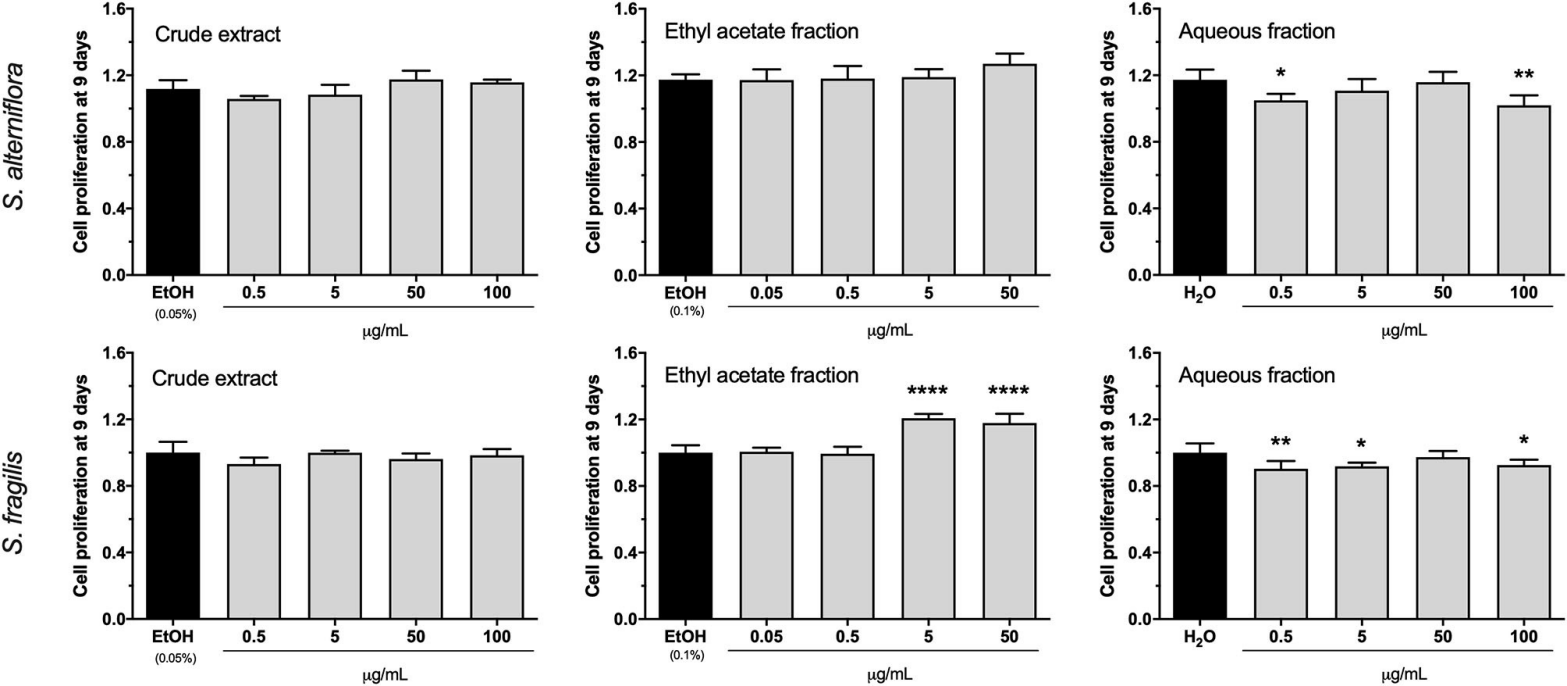
Proliferative activity of *S. alterniflora* (upper panel) and *S. fragilis* (lower panel) extracts *in vitro*. VSa13 cells were exposed for 9 days to the crude extracts (0.5, 5, 50, 100 μg/mL), ethyl acetate fractions (0.05, 0.5, 5, 50 μg/mL) or aqueous fractions (0.5, 5, 50, 100 μg/mL) of each plant, or to the respective vehicles (0.05% ethanol, 0.1% ethanol and water). Results are presented as fold-change over the control value (set to 1). Values are presented as mean ± standard deviation (*n* = 5). Asterisks indicate values significantly different from the control values (one-way ANOVA followed by Dunnett's multiple comparison test; *p < 0.05, **p < 0.01, ****p < 0.0001).

Proliferative activity was assessed in cell cultures exposed for 9 days to each of the 6 extracts and respective controls. For both species, CE did not alter cell proliferation at any of the concentrations tested and AF had a slight anti-proliferative effect at distinct concentrations. AF effect was not dose dependent and may be related with the reduced cell survival observed at 9 days (Supplementary Figure 1). This double effect was previously reported for this type of fraction (32) and would indicate that AF contains compounds that have a detrimental effect in the cells. While *S. alterniflora* EAF had no impact on cell proliferation, *S. fragilis* EAF showed a slight pro-proliferative effect (~20% increase over the control) at 5 and 50 μg/mL (Figure 1).

**Figure 2:**
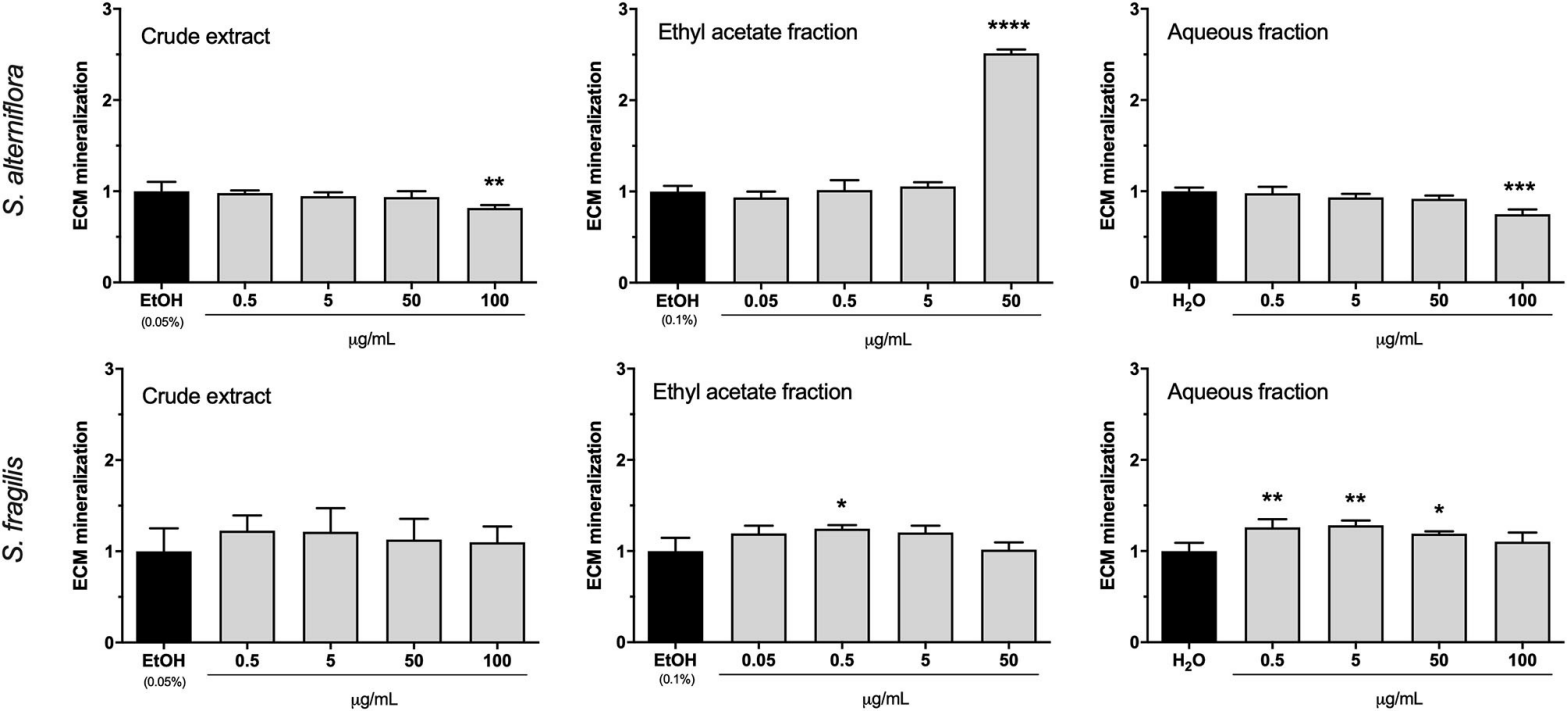
Mineralogenic activity of *S. alterniflora* (upper panel) and *S. fragilis* (lower panel) extracts *in vitro*. VSa13 cells were exposed for 17 days to the crude extracts (0.5, 5, 50, 100 μg/mL), ethyl acetate fractions (0.05, 0.5, 5, 50 μg/mL) or aqueous fractions (0.5, 5, 50, 100 μg/mL) of each plant, or to the respective vehicles (0.05% ethanol, 0.1% ethanol or water). Results are presented as fold-change over the control value (set to 1). Values are presented as mean ± standard deviation (*n* = 4). Asterisks indicate values significantly different from the control values (one-way ANOVA followed by Dunnett's multiple comparison test; *p < 0.05, **p < 0.01, ***p < 0.001, ****p < 0.0001).

Mineralogenic activity was assessed in cell cultures exposed for 17 days to each of the 6 extracts and respective controls. In general, CE had no impact on ECM mineralization although a small anti-mineralogenic activity was observed for the highest concentration of *S. alterniflora* CE (100 μg/mL; Figure 2), an effect that may be related with the cytotoxic effect previously observed at 18 days (Supplementary Figure 2). Similarly, *S. alterniflora* AF had no mineralogenic effect and only slightly decreased ECM mineralization at the highest concentration was observed, an effect that could be related to the anti-proliferative effect observed at this concentration (Figure 1). *S. fragilis* AF increased ECM mineralization by ~25% at concentrations ranging from 0.5 to 50 μg/mL but this pro-mineralogenic effect disappeared at higher concentrations for reasons that could be related to cytotoxic or antiproliferative effects previously observed. *S. alterniflora* EAF demonstrated a strong pro-mineralogenic capacity at 50 μg/mL, increasing ECM mineralization by 150% over the control. *In vitro* results evidenced the presence of mineralogenic molecules in *S. alterniflora* EAF. As it also contains antioxidant molecules, this extract was selected for further investigation.

### Validation of *Spartina alterniflora* EAF Bioactivies

**Figure 3:**
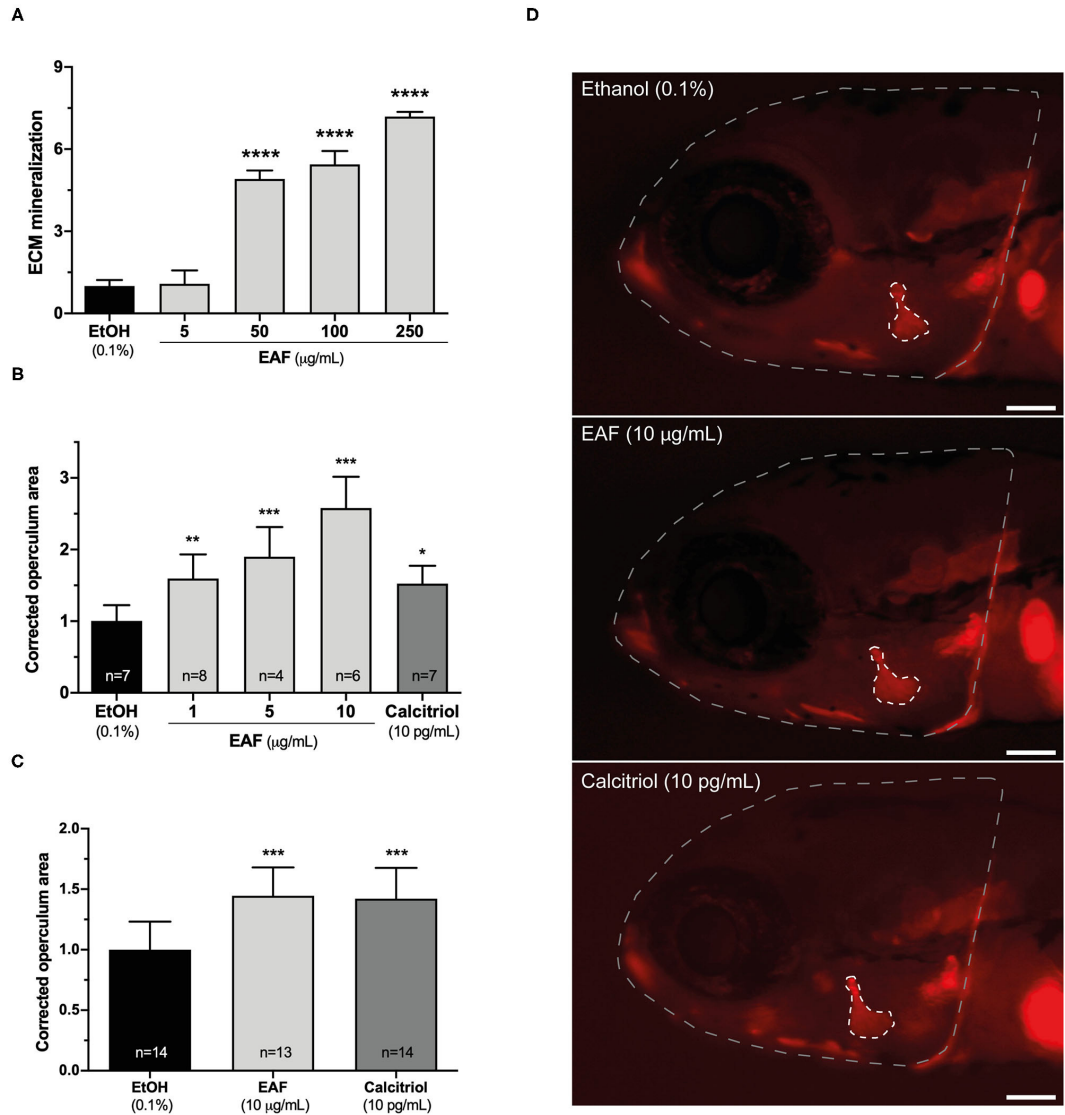
Mineralogenic and osteogenic activities of *S. alterniflora* ethyl acetate fraction. **(A)** EAF mineralogenic activity was determined in VSa13 cell cultures induced for ECM mineralization for 17 days while exposed to 5, 50, 100 and 250 μg/mL of the extract or 0.1% ethanol (*n* = 4). **(B,C)** EAF osteogenic activity was determined in 9-dpf zebrafish larvae exposed for 48 h to 1, 5, 10 μg/mL of EAF, 10 pg/mL of calcitriol or 0.1% ethanol (*n* > 4) **(B)** and in 3-dpf zebrafish larvae exposed for 72 h to 10 μg/mL of EAF, 10 pg/mL of calcitriol or 0.1% ethanol (*n* > 13) **(C)**. **(D)** Fluorescence images of alizarin red S-stained larvae exposed to the different conditions/treatments. Areas of the head and operculum are evidenced by dashed lines. White bar represents 100 μm. Values are presented as mean ± standard deviation. Asterisks indicate values significantly different from the control values (one-way ANOVA followed by Dunnett's multiple comparison test; *p < 0.05, **p < 0.01, ***p < 0.001, ****p < 0.0001).

To further explore the potential of *S. alterniflora* EAF as a source for bone anabolic compounds, the growth rate of the zebrafish opercular bone was determined in larvae exposed to the extract. Because this *in vivo* assay requires a large quantity of material, a novel extract was prepared from the same biomass and following the same methodology. Still, the antioxidant activity of the new EAF had first to be confirmed and an ORAC value of 1781.63 ± 356.33 μmol Te g^−1^ was measured. This value is in the same range as the one of vitamin C and is 10 times and 17 times higher than the ones for ginger root and black raspberry juice, respectively (Table 2), 3-well known natural antioxidants and used in the human diet (52, 53). This result not only confirms *S. alterniflora* EAF antioxidant properties, but also highlights its nutraceutical or pharmaceutical potential. Then the new extract was tested for its capacity to potentiate ECM mineralization, using an extended range of concentrations. i.e., 5, 50, 100, and 250 μg/mL. Concentrations repeated from the first evaluation (5 and 50 μg/mL) gave similar results, i.e., a strong mineralogenic effect at 50 μg/mL and none at 5 μg/mL, and ECM was further mineralized (up to 7 times over the control) at higher concentrations (100 and 250 μg/mL) (Figure 3A). Pro-mineralogenic effect of *S. alterniflora* EAF appeared to be dose dependent. Interestingly, no cytotoxic effect was associated with the highest concentration tested, i.e., 250 μg/mL (Supplementary Figure 3A) and higher concentrations may be tested in the future, with the possibility of having a stronger mineralogenic effect.

**Table 2:** Antioxidant activity of *S. alterniflora* ethyl acetate fraction determined by the ORAC test.

		**ORAC value (μmol Te g^**−1**^ DW)**
*S. alterniflora*	EAF	1,781.63 ± 356.33
Positive controls	Ascorbic acid^a^	9,350.00 ± 350.00
	Black raspberry juice^b^	104.60 ± 1.23
	Ginger root, raw^b^	148.40 ± 5.30

*The oxygen radical absorbance capacity (ORAC) of EAF is expressed as μmol Trolox equivalent (Te) g^−1^ dry weight and compared to ascorbic acid, black raspberry juice and ginger root*.

a *Huang et al. (53)*;

b *Haytowitz and Bhagwat (52)*.

Zebrafish larvae were also exposed to the new extract and the growth of the opercular bone was assessed through morphometrics (Figures 3B–D; Supplementary Figures 3B,C) and used as a proxy to evaluate the osteogenic potential of *S. alterniflora* EAF. First, 9-dpf larvae were exposed for 48 h to three concentrations (1, 5 and 10 μg/mL) and stained with AR-S. Image analysis revealed that operculum size was increased at all concentration tested (Figure 3B) and at levels comparable or higher than the positive control calcitriol (10 pg/mL), after normalization with the respective area of the head (Supplementary Figure 3B). Pro-osteogenic effect appeared to be dose-dependent and highest concentration more than doubled the size of the operculum. However, we observed some mortality at all concentrations tested, including the negative and positive control, a condition previously reported in similar experiments at this larval stage (49). To overcome this issue, the analysis was repeated using younger fish (larvae of 3 dpf) and a longer exposure (72 h) but also optimized husbandry conditions (49). Only the highest effective concentration was used, i.e., 10 μg/mL. Mortality was drastically reduced and *S. alterniflora* EAF could still significantly induce the growth of opercular bone (about 45%; Figure 3C) at a level comparable to that of the calcitriol. *In vivo* results evidenced the presence of osteogenic molecules in *S. alterniflora* EAF and further support its potential for the discovery of new anabolic compounds.

## Discussion

Following recent reports evidencing the presence of important bioactive compounds in marine organisms (32, 54–56), we investigated the halophytes *Spartina alterniflora* and *Salicornia fragilis* as natural sources of molecules with both antioxidant and pro-osteogenic activities. The preparation of the different extracts through liquid-liquid extraction proved to be effective and resulted in ethyl acetate fractions rich in phenolic compounds and exhibiting high antioxidant activities. Although the suitability of Folin-Ciocalteu test to measure phenolic compounds has been discussed due to the possible interference of proteins with aromatic hydroxylated rings (40), and a preliminary NMR spectra confirmed that aromatic compounds are present in EAFs (Supplementary Figure 4), this interference usually does not exceed 5% of the non-phenolic compounds that oxidize in this reaction (57). Such interference is even less if the protein residues are eliminated by precipitation in acetone during the purified process (36, 37), which is the case here. Therefore, the levels of phenolic compounds reported here can be considered reliable. Ethyl acetate is a solvent that has already been used to concentrate phenolic compounds (25, 58) and successfully applied to polyphenol enrichment in the EAF of other halophytes (22). Content in phenolic compounds determined in the CE of *S. alterniflora* and *S. fragilis* (21.96 ± 0.62 and 20.18 ± 0.36 mg GAE g^−1^ DW, respectively) are in the same order of magnitude as content measured in *Suaeda maritima* (23.5 ± 4.2 mg chlorogenic acid equivalent g^−1^ DW) (59). Moreover, the fractionation and/or purification of crude extracts seems to be important to concentrate phenolic compounds. In a study involving the characterization of 100 halophyte species, where no fractionation process was performed, plants extracts from the same family of *S. fragilis* and *S. alterniflora* (Amaranthaceae and Poaceae, respectively) had low levels of TPC (60). Rodrigues and colleagues reported that phenolic compounds were mainly concentrated in the methanol or diethyl ether fractions from five halophytes, but less abundant in hexane, chloroform and water fractions (54), while Surget et al. reported that phenolic compounds were efficiently concentrated in an ethyl acetate fraction of *S ramossissima* (22), further supporting our results.

Surprisingly, while flavonoids are highly representative of the phenolic compounds present in *S. fragilis* EAF (and to a lesser extent in CE and AF), they represented 50–60% of the TPC in *S. alterniflora* CE and AF, although these compounds could not be detected in the EAF due to interferences. Previous studies focusing on crude extracts found that *S. alterniflora* leaf extracts contained mainly flavonoids (61, 62), with TPC levels similar to those presented here for CE and AF, supporting our findings. Ethanolic extracts prepared from three halophytes of the Poaceae family were found to contain amounts of phenolic compounds similar to those reported here; these extracts, including one prepared from *Spartina maritima*, also contained flavonoids (63). While few studies have characterized TPC in extracts of *Spartina* species, the presence of flavonoids and phenolic acids in the genus *Salicornia* has been reported in several studies (22, 64, 65). Among those, Surget et al. demonstrated that *Salicornia ramossissima* from Brittany, as the species under study *S. fragilis*, produced several flavanols and caffeic acid derivatives (22). These and other studies highlighted the relevance of their phytochemical profile on the numerous medicinal effects reported for these edible plants (66–68).

Several studies have associated the content of phenolic compounds in plant extracts with their antioxidant activities (22, 54, 60, 69–72). Our data confirmed that the fractions with the highest content in TPC were also the fractions with the highest antioxidant activities. Data collected for *S. fragilis* are supported by previous studies that has extensively characterized antioxidant activities in *Salicornia* species through similar or different *in vitro* tests (22, 66, 68, 73, 74). Qasim et al. reported levels of DPPH IC_50_ similar to those reported here for CE and AF in extracts from several species of the Amaranthaceae and Poaceae families, in accordance with their low content of phenolic compounds as well (60). In another study, DPPH scavenging activity of extracts prepared from two *Spartina* species was evaluated with comparable results to those reported here for *S. alterniflora* CE and AF (63). *S. fragilis* EAF exhibited reducing activities (EC_50_ of 0.239 g/L) similar to those determined in the halophytes *Tamarix gallica* and *Salsola kali*, 0.205 and 0.458 g/L, respectively (21). On the contrary, β-carotene bleaching test revealed low levels of antioxidant activity in both species, a result that may be related to the polarity of the extracts. Indeed, apolar antioxidants show stronger activities in emulsion because they are concentrated at the linoleic acid/air interface, thus protecting the emulsion from oxidation. Polar antioxidants, on the other hand, show weaker activities because they do not concentrate at this interface (40), as it is the case for phenolic acids (42).

Following evidences indicating that natural compounds with antioxidant properties have a positive impact on bone metabolism (9, 17, 75, 76) and could be used to prevent or treat bone diseases such as osteoporosis (8, 9, 11, 12), we evaluated the bone anabolic properties of the phytochemicals present in the fractions with high TPC and antioxidant activities. Although *S. fragilis* EAF had the highest content in flavonoids, it only triggered a weak mineralogenic effect; on the contrary, *S. alterniflora* EAF strongly induced ECM mineralization in VSa13 cell cultures suggesting the presence in this fraction of compounds with the ability to promote osteoblast differentiation but not proliferation. Other studies have reported an anabolic effect of phenolic compounds, and in particular flavonoids, on osteoblasts (33, 77). Extracts from *Salicornia herbacea* (Amaranthaceae) promoted osteoblastogenesis in differentiating MC3T3 cells by increasing alkaline phosphatase (ALP) activity and up-regulating the expression of bone marker genes, while inhibiting adipogenesis (78). *Achyranthes bidentata* (Amaranthaceae), a plant widely used in Chinese herbal medicine, has also been shown to contribute for the proper function of osteoblasts (79). All these studies clearly evidence the presence of compounds with the ability to promote osteoblast function in halophytes of the Amaranthaceae family, further supporting our data demonstrating the presence of osteoactive molecules in *S. alterniflora* EAF extract.

*S. alterniflora* EAF antioxidant activity was corroborated by the ORAC assay which uses the most predominant free radical in human physiology (peroxyl radical), thus evidencing its relevance in *in vivo* conditions. The ORAC value for *S. alterniflora* EAF indicated a good antioxidant activity when compared to the extracts of plants such as *Moringa oleifera* (80) but also vitamin C, ginger root and black raspberry juice, well-known antioxidants used in the human diet (52, 53), highlighting the interest to isolate bioactive phenolic compounds from halophytes in order to supplement some dietary regimes. The conserved bioactivity in *S. alterniflora* new EAF fraction is a clear indication that the extraction process can led to a reproducible enrichment in osteoactive and antioxidant compounds. Importantly, cell survival was not affected in cultures exposed to high EAF concentrations, indicating the absence (or the presence in very limited amount) of cytotoxic compounds in this fraction, an important aspect if an application for human health is foreseen. *S. alterniflora* EAF was further tested using zebrafish as it presents several advantages when compared to mammalian models, in particular for the study of osteogenesis and bone-related diseases such as osteoporosis (81). The possibility of imaging bone structures while they form is one of the benefits associated with translucent zebrafish larvae, and the operculum system used in this work (49) has already been successfully applied in several studies to uncover osteogenic properties of extracts from different marine species (30–32). An increased bone formation was observed in zebrafish larvae exposed to *S. alterniflora* EAF and we propose, based on *in vitro* data, that this bone anabolic effect is triggered by the stimulation of osteoblast differentiation, thus their osteogenic capacity, rather than proliferation. We also hypothesize that mineralogenic and osteogenic activities of *S. alterniflora* EAF are related to its high phenolic content and antioxidant activity. In this regard, the ethanolic extract of *Cynodon dactylon* (Poaceae) showed a significant improvement of bone lesions (82), and an attenuation of the inflammatory response and oxidative stress (83) in rats with adjuvant-induced arthritis, further evidencing a link between antioxidant and bone-related effects. The hypothesis that antioxidant compounds could be effective candidates for the prevention of bone diseases such as osteoporosis by restraining bone loss through the inhibition of oxidative stress has been reported in the literature by different authors (8, 9, 17, 33, 84, 85). Furthermore, polyphenols were also recognized as beneficial on bone health, especially for people suffering osteoporosis (86). Accordingly, extracts from other Poaceae plant, the common bamboo (*Bambusa vulgaris*), could improve bone calcium concentration and femur microarchitecture in an osteoporotic rat model to levels similar to those achieved by 17β-estradiol treatment, suggesting a therapeutical potential for osteoporosis (87). Also, bioactive extracts/polyphenols could be included in biomaterials used for tissue transplants to help prevent inflammation while boosting bone production as described for other marine polyphenols (88).

## Conclusions

The number of studies aiming at the discovery of natural compounds with pharmaceutical or nutraceutical value for bone health and/or bone healing has largely increased over the last decades (14, 89, 90). This recent interest in the search for alternative drugs further evidences the yet unmet need for effective preventive and/or therapeutic strategies for bone diseases while highlighting the value of marine organisms as natural sources of novel bioactive compounds (18, 22, 30, 32). Our data provides new perspectives in this field as few studies have reported the bone anabolic effect of phenolic compounds-rich fractions from halophytic organisms. As an invasive species found in salt marshes of many countries in the world, *Spartina alterniflora* would represent a sustainable source of molecules for therapeutic applications. The phenolic compounds present in the ethyl acetate fraction of this marine plant are thus potential candidates to join the class of marine phenolic compounds for alternative anti-osteoporotic therapies.

## Data Availability Statement

The original contributions presented in the study are included in the article/Supplementary Material, further inquiries can be directed to the corresponding author/s.

## Ethics Statement

The animal study was reviewed and approved by ORBEA, UALG Ethical Committee.

## Author Contributions

VR, GS, KL, SM, MT, FG, NP, VL, VS-P, and MC: conceptualization. VR, SM, VL, MT, GS, and VS-P: methodology. VR, SM, and MT: validation. GS, VS-P, VR, and VL: formal analysis. FG, VS-P, NP, and MC: investigation and resources. VR: writing—original draft preparation. VS-P, FG, NP, MC, and VL: writing—review and editing. VR and VL: visualization. VS-P: supervision. FG, VS-P, NP, MC, and VL: project administration and funding acquisition. All authors contributed to the article and approved the submitted version.

## Conflict of Interest

The authors declare that the research was conducted in the absence of any commercial or financial relationships that could be construed as a potential conflict of interest.

## Publisher's Note

All claims expressed in this article are solely those of the authors and do not necessarily represent those of their affiliated organizations, or those of the publisher, the editors and the reviewers. Any product that may be evaluated in this article, or claim that may be made by its manufacturer, is not guaranteed or endorsed by the publisher.
