# Supplementary material for: Antioxidant, Mineralogenic and Osteogenic Activities of Spartina alterniflora and Salicornia fragilis Extracts Rich in Polyphenols
Source: Front Nutr. 2021 Aug 18;8:719438. doi: 10.3389/fnut.2021.719438 (PMC8416452; doi:10.3389/fnut.2021.719438)
Supplement: Supplementary file 1 [file Data_Sheet_1.PDF]

Supplementary Material

1.1 Supplementary Figures

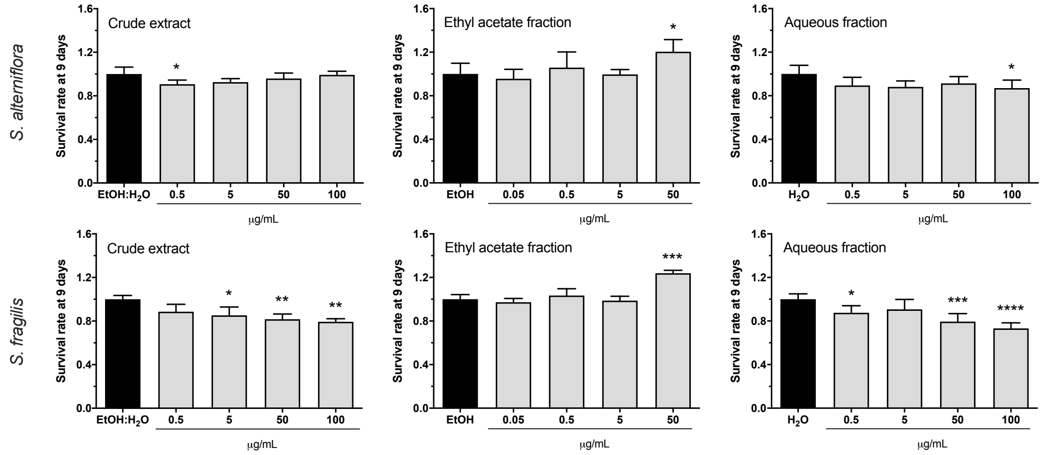

Supplementary Figure S1. Survival of VSa13 cells exposed for 9 days to *S. alterniflora* (upper panel) and *S. fragilis* (lower panel) extracts *in vitro*. Results are presented as fold-change over the control value (set to 1). Values are presented as mean ± standard deviation (n=5). Asterisks indicate values significantly different from the control values (one-way ANOVA followed by Dunnett's multiple comparison test; *p < 0.05, **p < 0.01, ***p < 0.001, ****p < 0.0001).

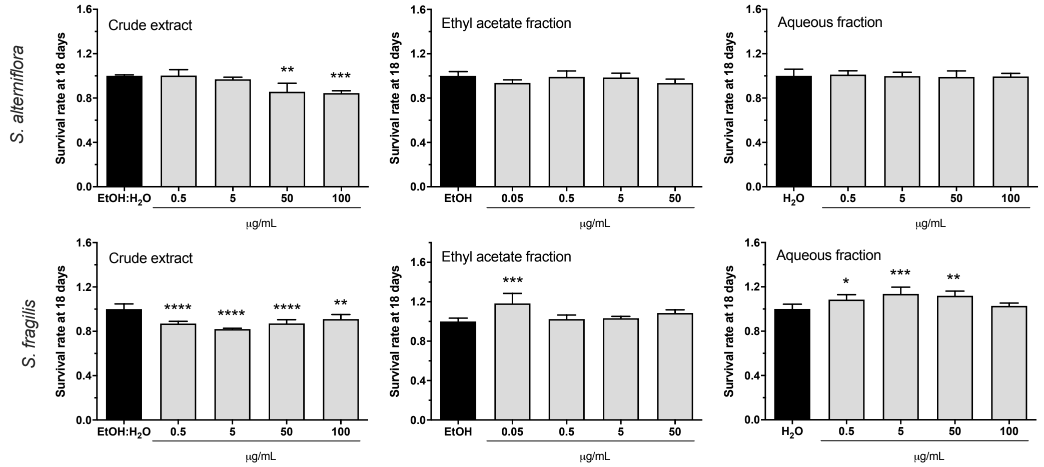

Supplementary Figure S2. Survival of VSa13 cells exposed for 18 days to *S. alterniflora* (upper panel) and *S. fragilis* (lower panel) extracts *in vitro*. Results are presented as a fold-change over the control value (set to 1). Values are presented as mean ± standard deviation (n=5). The asterisks indicate values significantly different from the control value (one-way ANOVA followed by Dunnett's multiple comparison test; *p < 0.05, **p < 0.01, ***p < 0.001, ****p < 0.0001).

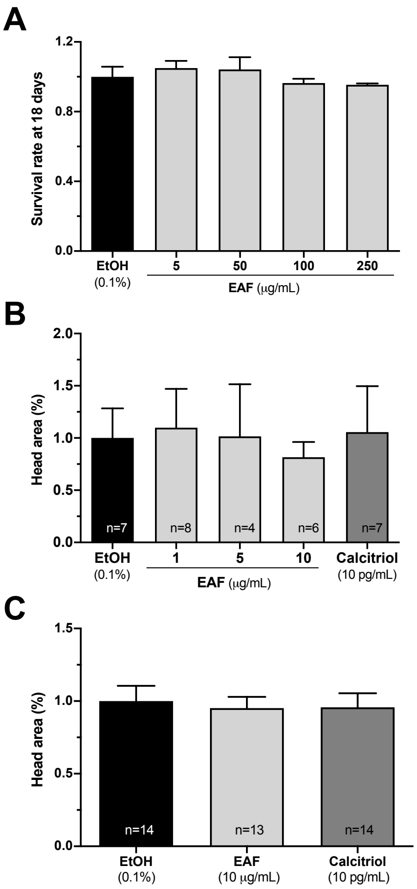

Supplementary Figure S3. Characterization of the novel EAF of *S. alterniflora*. (**A**) Survival of VSa13 cells exposed for 18 days to 5, 50, 100 and 250 µg/mL of EAF. Results are presented as fold-change over the control value (set to 1) (n=5). (**B-C**) The area of the head of 9-dpf zebrafish larvae exposed for 48 h to 1, 5 and 10 µg/mL of EAF, 10 pg/mL of calcitriol or 0.1% ethanol (n>4) (**B**) and of 3-dpf zebrafish larvae exposed for 72 h to 10 µg/mL of EAF, 10 pg/mL of calcitriol or 0.1% ethanol (n>13) (**C**). The area of the head was used to normalized the area of the operculum. Results are presented as fold-change over the control value (set to 1).Values are presented as mean ± standard deviation. No significant differences were found (one-way ANOVA).

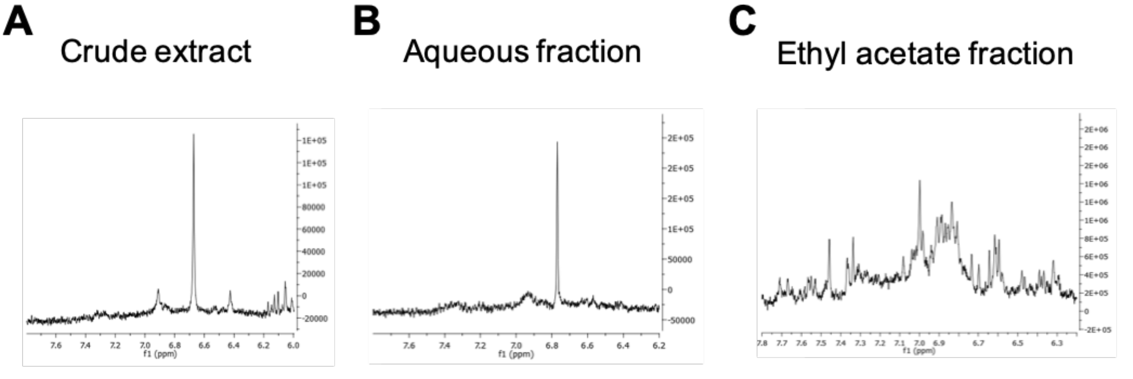

Supplementary Figure S4. ^{1}H NMR spectra of crude extracts and semi-purified fractions of *S. alterniflora*. The spectra are zoomed in on the 6 to 8 ppm region. **A**) The crude extract and **B**) the aqueous fraction were solubilized in deuterium oxide (D2O), while **C**) the ethyl acetate fraction in deuterated methanol. Chemical shifts (x-axis) are in ppm.
